# Early lymphocyte recovery predicts cardiovascular events and mortality in immune checkpoint inhibitor-associated myocarditis: a retrospective cohort study

**DOI:** 10.3389/fcvm.2026.1870541

**Published:** 2026-07-22

**Authors:** Bin Zhang, Guangcheng Liu, Kanghao Zhou, Xiong Zhang, Yue Fan, Yan Xu, Wei Wu

**Affiliations:** 1 Department of Cardiology, Peking Union Medical College Hospital, Chinese Academy of Medical Sciences and Peking Union Medical College, Beijing, China; 2 Department of Respiratory and Critical Care Medicine, Peking Union Medical College Hospital, Chinese Academy of Medical Sciences and Peking Union Medical College, Beijing, China

**Keywords:** absolute lymphocyte count, cardio-oncology, immune checkpoint inhibitor-associated myocarditis, major adverse cardiovascular events, overall survival, risk stratification

## Abstract

**Background:**

Immune checkpoint inhibitor-associated myocarditis is an uncommon but potentially fatal cardiovascular toxicity of cancer immunotherapy. Early risk stratification remains challenging, and readily available biomarkers are needed. We investigated whether early changes in absolute lymphocyte count (ALC) were associated with major adverse cardiovascular events (MACE) and overall survival in patients with immune checkpoint inhibitor-associated myocarditis.

**Methods:**

This retrospective cohort study included 60 patients diagnosed with immune checkpoint inhibitor-associated myocarditis. Absolute lymphocyte count was measured serially during the first 9 days after corticosteroid initiation. A Day-9 landmark design was used to assess associations between lymphocyte dynamics and subsequent outcomes. The primary endpoint was 60-day major adverse cardiovascular events, and the secondary endpoint was 1-year overall survival. Cox regression, Kaplan–Meier analysis, receiver operating characteristic analysis, and restricted cubic spline modeling were performed.

**Results:**

Among 60 patients, 28 (46.7%) developed MACE in 60 days. Patients who developed MACE had persistently lower ALC levels and impaired early lymphocyte recovery. Day-9 ALC demonstrated good discrimination for MACE (AUC = 0.816) and remained independently associated with lower risk after multivariable adjustment (HR 0.33, 95% CI 0.14–0.81; *p* = 0.016). The association between Day-9 ALC and MACE risk was nonlinear, with lower estimated risk at higher ALC levels. Faster lymphocyte recovery was also independently associated with lower MACE risk (HR 0.07 per 0.1 × 10^9^/L/day, 95% CI 0.01–0.44; *p* = 0.004). Both lower Day-9 ALC and impaired recovery were associated with worse 1-year overall survival.

**Conclusions:**

In patients with immune checkpoint inhibitor-associated myocarditis, lower Day-9 absolute lymphocyte count and slower early lymphocyte recovery were associated with adverse cardiovascular events and mortality. Serial absolute lymphocyte count assessment may provide a simple and clinically accessible approach for early risk stratification, although external validation is needed.

## Introduction

Immune checkpoint inhibitors (ICIs) have profoundly improved outcomes across a wide range of cancer therapy. Meanwhile, the expanding usage of ICIs can cause a variety of immune-related adverse events (irAEs) that pose substantial clinical challenges. Immune checkpoint inhibitor-associated myocarditis (ICIAM) is an uncommon type of irAEs, with an incidence reported to range from 0.27% to 1.14% (1, 2). ICIAM is notable for early onset of 21–34 days after ICIs-treatment initiation, rapid progression, high risk of major cardiovascular complications and high mortality of 38%–50% (1, 3–5). Existing risk stratification in ICIAM has focused mainly on cardiac biomarkers, ECG findings, and imaging abnormalities (6–9). Although recognition of this entity has improved, early risk stratification remains difficult in routine practice, and biomarkers that can help identify patients at increased risk of adverse outcomes are still needed (10, 11).

Given that ICIAM is characterized by immune dysregulation and T-cell–mediated myocardial injury, there is increasing interest in identifying clinically accessible immune-related biomarkers that can be monitored dynamically during treatment. Absolute lymphocyte count (ALC) is inexpensive, routinely available, and may offer a practical means of serial immune monitoring (2). Previous work has suggested that lymphocyte-related parameters such as decreased ALC may be associated with worse clinical outcomes in ICIAM. However, prior studies have primarily focused on baseline or single time-point hematologic parameters, and the clinical relevance of early ALC change has not been well defined (12, 13). Furthermore, dynamic changes in circulating lymphocytes may provide prognostic information beyond a single baseline measurement. Interpretation is further complicated by corticosteroid therapy, the cornerstone of first-line treatment, because steroids can substantially alter circulating lymphocyte counts during the acute phase (14, 15).

Accordingly, whether impaired lymphocyte recovery reflects only treatment-related hematologic fluctuation or instead represents a clinically meaningful signal of persistent immune dysregulation remains uncertain (15, 16). This question is especially important because ICIAM is frequently complicated by major adverse cardiovascular events (MACE), and emerging data suggest that early clinical and biomarker responses may also be relevant to longer-term survival (13, 17). In routine clinical practice, early identification of high-risk patients remains a key clinical challenge.

Therefore, in this study, we characterized early dynamic changes in ALC in patients with ICIAM and investigated whether early lymphocyte dynamics can enable clinically meaningful risk stratification for MACE and 1-year overall survival.

## Materials and methods

### Study design and population

We conducted a retrospective study of 60 patients diagnosed with ICIAM at Peking Union Medical College Hospital between January 2023 and January 2025. Data were extracted from the hospital health information system, including demographics; cancer diagnosis; prior or concomitant chemotherapy and targeted therapy; type of ICI; comorbidities; smoking and alcohol history; baseline laboratory results, including hemoglobin (HGB), white blood cells (WBC), absolute neutrophil count (ANC), absolute lymphocyte count (ALC), monocytes (MONO), platelets (PLT), albumin (Alb); baseline cardiac biomarkers [cardiac troponin I (cTnI) and N-terminal pro–B-type natriuretic peptide (NT-proBNP)]; corticosteroid therapy, immunosuppressants, and intravenous immunoglobulin (IVIG); and clinical outcomes. Serial ALC values were retrospectively extracted from routine complete blood count tests performed during hospitalization. Day 0 was defined as the time of corticosteroid initiation, and ALC values at Day 0, Day 3, Day 6, and Day 9 were obtained from routine clinical laboratory records. cTnI was expressed as multiples of the upper limit of normal (ULN). NT-proBNP was log-transformed due to its extreme values and improve model stability. To reduce bias related to corticosteroid exposure and time-dependent treatment effects, we standardized corticosteroid exposure by defining 40 mg/day intravenous methylprednisolone (or 50 mg/day oral prednisone) as 1 “dose-equivalent” unit. Cumulative dosages were calculated for day 0–3 (D0–3), day 3–6 (D3–6), day 6–9 (D6–9), and overall day 0–9 (D0–9).

**Figure 1:**
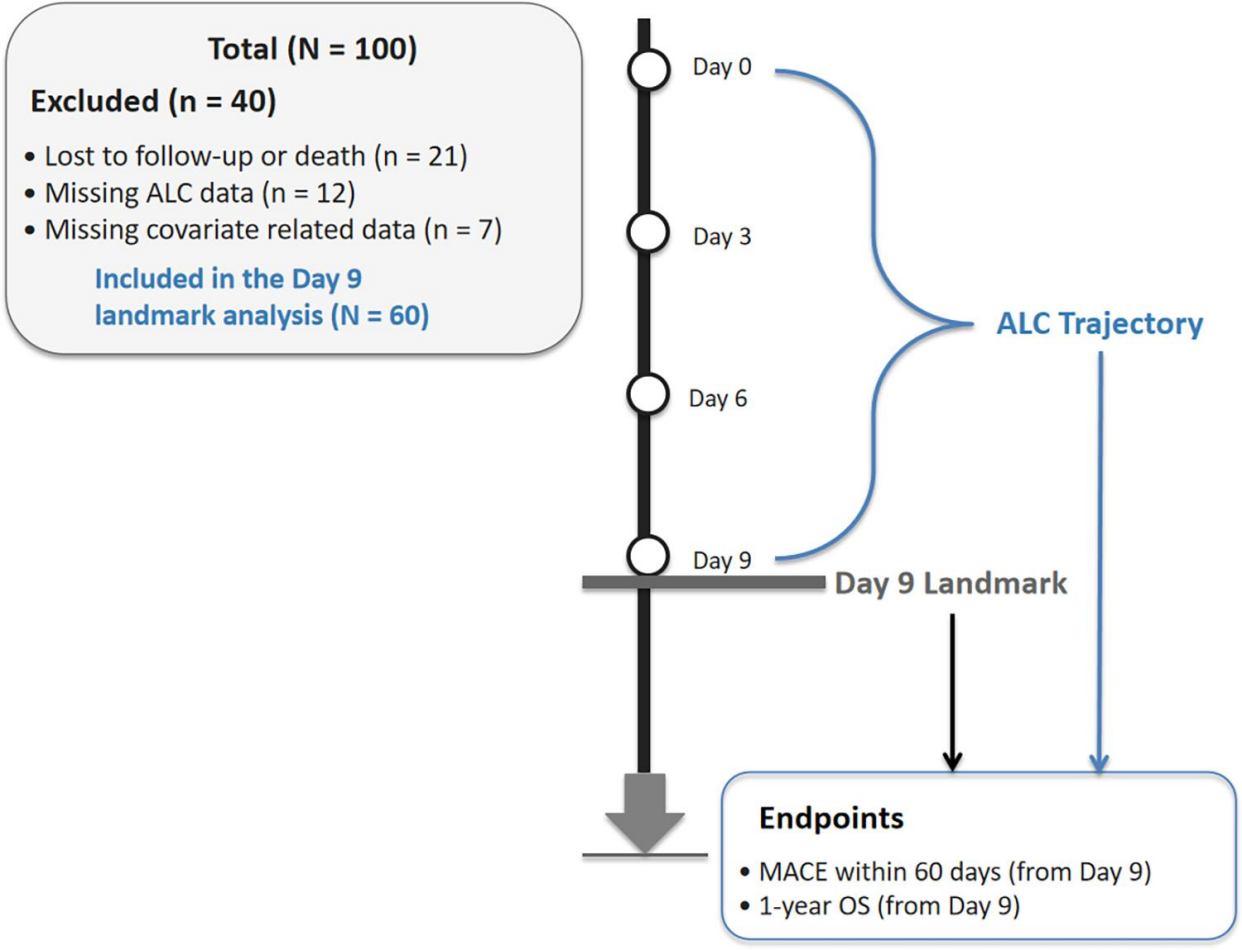
Study flowchart. Flowchart of patient screening, eligibility assessment, and inclusion in the final retrospective cohort of patients with immune checkpoint inhibitor–associated myocarditis. Reasons for exclusion are shown in the flowchart.

The study flowchart is shown in Figure 1. Patients were eligible if they were aged 18 years or older, had received at least one dose of an immune checkpoint inhibitor, fulfilled the diagnostic criteria for ICIAM, and had serial ALC measurements available during the first 9 days after corticosteroid initiation. Patients were excluded if they had insufficient clinical records to confirm the diagnosis or outcomes, missing key follow-up information before Day 9, or pre-existing hematologic conditions that substantially affected lymphocyte counts. And no imputation was performed. Patients who experienced MACE before the Day-9 landmark were excluded from the landmark survival analyses.

ICIAM was diagnosed according to the 2022 European Society of Cardiology cardio-oncology guideline, integrating clinical presentation, cardiac biomarkers, imaging, or pathology (18). Cardiovascular magnetic resonance (CMR) diagnosis followed the 2018 Lake Louise criteria (19). Endomyocardial biopsy findings were adjudicated by Dallas criteria (20). Final clinical diagnoses were made by cardiologists based on biopsy findings, CMR, biomarker elevation, symptoms, and other clinical features. Corticosteroid therapy was administered in accordance with the 2022 European Society of Cardiology cardio-oncology guidelines (18). Patients with severe presentations received initial high-dose corticosteroids, whereas those with less severe disease were treated with moderate-dose regimens. Subsequent tapering and maintenance therapy were performed according to clinical response and physician decision. The 3-day interval was clinically chosen because corticosteroid pulse therapy at our institution is commonly administered for approximately 3 days, followed by step-down treatment and reassessment at similar intervals.

### Study Endpoints

The primary endpoint was the occurrence of MACE, defined—consistent with prior ICI studies—as a composite of cardiac death, cardiac arrest, cardiogenic shock, sustained ventricular tachyarrhythmia, and hemodynamically significant atrioventricular block. Deaths attributable to cardiac arrest, cardiogenic shock, ventricular tachyarrhythmia or complete atrioventricular block were counted as cardiac deaths. For patients with multiple MACE, time to first event was used. MACE-free survival time was measured from Day 9 after corticosteroid initiation to the first occurrence of MACE, with follow-up censored at 60 days. The secondary endpoint was 1-year overall survival, defined as the time from Day 9 after corticosteroid initiation to death from any cause.

### Statistical Analysis

Categorical variables are presented as counts and percentages, and continuous variables as mean ± standard deviation or median [interquartile range (IQR)], as appropriate. Between-group comparisons for categorical variables were performed using the *χ*² test or Fisher's exact test, as appropriate. For continuous variables, normality was assessed using the Shapiro–Wilk test; comparisons between two groups were performed using the independent-samples t-test for normally distributed data and the Mann–Whitney U test otherwise. For analyses involving exposures derived from Day 0–9 absolute lymphocyte count (ALC), a Day-9 landmark design was used, with follow-up re-originated at Day 9 to reduce immortal time bias. Between-group differences in ALC at each time point were compared using the Mann–Whitney U test, with Bonferroni correction for multiple comparisons across the four time points. Within-group changes in ALC across serial time points (Day 0, Day 3, Day 6, and Day 9) were assessed separately in the MACE and no-MACE groups using the Friedman test, followed by Bonferroni-adjusted *post hoc* comparisons. Receiver operating characteristic (ROC) analysis was used to determine the optimal Day-9 ALC cutoff according to Youden's index. Kaplan–Meier curves were constructed and compared using the log-rank test to examine associations between ALC-based strata and MACE-free survival. Univariable and multivariable Cox proportional hazards models were used to evaluate the association of Day-9 ALC with outcomes. Given the limited number of events, the multivariable model adjusted for age, sex, left ventricular ejection fraction, ln(NT-proBNP), and steroid pulse therapy. The log-transformed NT-proBNP variable was used in Cox regression models to better approximate a linear relationship with the log hazard. No automated variable selection procedure was used. The proportional hazards assumption was assessed using Schoenfeld residuals. To explore potential nonlinearity, Day-9 ALC was modeled in the multivariable Cox model using restricted cubic splines. To further characterize dynamic lymphocyte recovery, each patient's overall Day 0–9 ALC change rate was estimated using linear mixed-effects models and summarized as an individual recovery slope. These slopes were then examined in multivariable Cox regression analyses; for interpretability, slopes were rescaled per 0.1 × 10⁹/L/day. For categorical risk stratification, a prespecified fixed equivalence band of ±0.01 × 10⁹/L/day was used to represent minimal overall change. Over the 9-day observation period, this corresponds to an approximate cumulative change of 0.1 × 10⁹/L. Patients were therefore classified as Falling (< −0.01 × 10⁹/L/day), Stable (−0.01–0.01 × 10⁹/L/day), or Rising (> 0.01 × 10⁹/L/day), and Kaplan–Meier curves were compared across groups using the log-rank test. All tests were two-sided, and *P* < 0.05 was considered statistically significant. Statistical analyses were performed using R version 4.2.

## Results

### Baseline characteristics, clinical presentation, and treatment

Among 60 patients with ICIAM, 28 (46.7%) developed MACE, and baseline characteristics are summarized in Table 1. Among patients with MACE, the most frequent component was cardiogenic shock (11/28, 39.3%), followed by ventricular tachyarrhythmias (9/28, 32.1%), high-grade atrioventricular block (8/28, 28.6%), and cardiac death (6/28, 21.4%). Cardiac arrest occurred in 2 patients (7.1%). The mean age of the cohort was 65 ± 9 years. There were no statistically significant differences between the MACE and no-MACE groups in age, comorbidities, cancer types, or baseline laboratory values (WBC, ANC, ALC, MONO, PLT, Alb). Hemoglobin was modestly higher in the MACE group than in the no-MACE group (130 ± 20 vs. 118 ± 21 g/L; *p* = 0.023). Most patients received PD-1 inhibitors (86.7%); 68.3% and 31.7% had received chemotherapy and targeted therapy, respectively. The median time from first ICI exposure to onset of myocarditis was 31 days (IQR, 22–58); this interval was shorter in the MACE group than in the no-MACE group [23 days (IQR, 18–46) vs. 41 days (IQR, 27–69)]. The most common presenting symptom was chest tightness (63%), followed by fatigue (45%), dyspnea (43%), and ptosis (31%). Chest tightness (78%) and dyspnea (57%) were more frequent in the MACE group than the no-MACE group (50% and 31%, respectively).

**Table 1:** Basic characteristics of patients with immune checkpoint inhibitor-associated myocarditis.

Patient characteristics	Overall (*n* = 60)	MACE (*n* = 28)	No-MACE (*n* = 32)	*P*-value
Age, year^a^	65 (9)	67 (10)	64 (8)	0.203
Male, *n* (%)	43 (71.7)	21 (75.0)	22 (68.8)	0.592
BMI^a^	23.8 (3.5)	23.2 (3.5)	24.3 (3.4)	0.234
Comorbidities, *n* (%)				
Coronary artery disease	12 (20.0)	4 (14.3)	8 (25.0)	0.301
Hypertension	35 (58.3)	19 (67.9)	16 (50.0)	0.162
Diabetes mellitus	20 (33.3)	10 (35.7)	10 (31.3)	0.714
History of smoking	33 (55.0)	14 (50.0)	19 (59.4)	0.466
History of drinking	19 (31.7)	6 (21.4)	13 (40.6)	0.111
Cancer type, *n* (%)				0.927
Gastric tumor	18 (30.0)	8 (28.6)	10 (31.3)	
Lung cancer	25 (41.7)	11 (39.3)	14 (43.8)	
Liver cancer	7 (11.7)	4 (14.3)	3 (9.4)	
Other	10 (16.7)	5 (17.9)	5 (15.6)	
Cancer treatment, *n* (%)				0.541
PD-1	52 (86.7)	25 (89.3)	27 (84.4)	
PD-L1	4 (6.7)	1 (3.6)	3 (9.4)	
PD-1 + CTLA-4	4 (6.7)	2 (7.1)	2 (6.3)	
Chemotherapy	41 (68.3)	19 (67.9)	22 (68.8)	0.941
Targeted therapy	19 (31.7)	9 (32.1)	10 (31.3)	0.941
Symptom, *n* (%)				
Chest tightness	38 (63.3)	22 (78.6)	16 (50.0)	**0**.**022**
Ptosis	19 (31.7)	11 (39.3)	8 (25.0)	0.235
Dyspnea	26 (43.3)	16 (57.1)	10 (31.3)	**0**.**043**
Fatigue	27 (45.0)	13 (46.4)	14 (43.8)	0.835
Palpitations	13 (21.7)	6 (21.4)	7 (21.9)	0.967
First ICIs to Symptom, days^b^	31 (22–58)	23 (18–46)	41 (27–69)	**0**.**003**
Baseline blood parameters				
Hemoglobin, (g/L)^a^	123 (22)	130 (20)	118 (21)	**0**.**023**
White blood cell count, (× 10^9^/L)^b^	6.74 (5.39–8.33)	7.16 (5.69–9.82)	6.33 (5.10–8.18)	0.134
Absolute neutrophil count, (× 10^9^/L)^b^	6.21 (5.19–8.28)	6.62 (5.42–9.03)	5.64 (4.42–7.03)	0.054
Absolute lymphocyte count, (× 10^9^/L)^b^	0.90 (0.69–1.18)	0.84 (0.67–1.11)	0.96 (0.72–1.26)	0.200
Absolute monocyte count, (× 10^9^/L)^b^	0.45 (0.29–0.66)	0.47 (0.26–0.56)	0.43 (0.31–0.68)	0.514
Platelets, (× 10^9^/L)^a^	208 (82)	199 (91)	215 (74)	0.446
Alb (g/L)^a^	37.8 (4.7)	36.9 (3.9)	38.7 (5.3)	0.139
Cardiac biomarkers				
NTproBNP (pg/mL)^b^	749 (157–1,714)	1,112 (693–2,376)	271 (97–1,215)	**0**.**002**
LnNTproBNP^a^	6.34 (1.49)	7.00 (1.12)	5.77 (1.56)	**<0**.**001**
cTnI (ULN times)^b^	17.2 (8.3–45.6)	21.7 (11.1–48.2)	12.0 (6.3–43.7)	0.082
LVEF^b^	64 (56–72)	57 (40–65)	68 (61–73)	**<0**.**001**

Continuous variables are presented as mean ± SD or median (IQR), and categorical variables as *n* (%).

Bold values indicate statistical significance (*P* < 0.05).

a indicates mean ± SD.

b indicates median (IQR).

Nearly all patients had elevated cTnI, with a median peak of 21× the upper limit of normal (ULN) (IQR, 9–57×). The median NT-proBNP level was 749 pg/mL (IQR, 157–1,714 pg/mL), and levels were higher in the MACE group than in the no-MACE group. Electrocardiography showed ventricular tachycardia or ventricular fibrillation in 10 patients, high-grade atrioventricular block in 12, and ST-T abnormalities in 15. On transthoracic echocardiography, the median left ventricular ejection fraction (LVEF) was 64% (IQR, 56%–72%), and 10 patients (17%) had LVEF <50%. Baseline LVEF was lower in the MACE group than in the no-MACE group [median, 57% (IQR, 40%–65%) vs. 68% (IQR, 61%–73%)]. Regional wall-motion abnormalities were present in 17 (29%) patients, and diastolic dysfunction in 30 (50%). Cardiovascular magnetic resonance (CMR) was performed in 34 patients (58%). Among these patients, 24 had positive CMR findings, accounting for 70% of patients who underwent CMR and 40% of the overall cohort. Endomyocardial biopsy was performed in 18 patients (30%), typically showing interstitial or interfascicular T-lymphocytic infiltration.

Treatment patterns and corticosteroid exposure differed between groups, and serial absolute lymphocyte count measurements across time points are summarized in Table 2. All patients received corticosteroids after ICIAM was diagnosed; 25 (41%) received steroid pulse therapy, 22 (36%) received additional immunosuppressants, and 45 (75%) received IVIG. Pulse therapy (64% vs. 21%) and IVIG use (96% vs. 56%) were more common in the MACE group than in the no-MACE group. Regarding steroid exposure, the MACE group received a substantially higher dose-equivalent during D0–3 than the no-MACE group [12.5 (IQR, 5–25) vs. 3 (IQR, 2–8), *p* < 0.001], as well as a higher cumulative dose-equivalent over D0–9 [18.5 (IQR, 12–28) vs. 7.5 (IQR, 4–13), *p* < 0.001]; no between-group differences were observed for D3–6 or D6–9.

**Table 2:** Treatment characteristics and serial absolute lymphocyte count measurements.

Variables	Overall (*n* = 60)	MACE (*n* = 28)	No-MACE (*n* = 32)	*P*-value
Treatment *n* (%)				
Steroid pulse therapy	25 (41.7)	18 (64.3)	7 (21.9)	**<0** **.** **001**
Immunosuppressants	22 (36.7)	10 (35.7)	12 (37.5)	0.717
IVIG	45 (75.0)	27 (96.4)	18 (56.3)	**<0**.**001**
Steroid Dosage				
Steroid dosage D0-D3 (times)^a^	6 (2–12.5)	12.5 (5–25)	3 (2–8)	**<0**.**001**
Steroid dosage D3-D6 (times)^a^	2 (1.5–3.75)	2 (2–4)	2 (1–3)	0.476
Steroid dosage D6-D9 (times)^a^	1 (1–2)	2 (1–2)	1 (1–2)	0.088
Total steroid dosage D0-D9 (times)^a^	11 (5–20.25)	18.5 (12–28)	7.5 (4–13)	**<0**.**001**
Absolute lymphocyte count, (× 10^9^/L)				
Absolute lymphocyte count, D0^a^	0.90 (0.69–1.18)	0.84 (0.67–1.11)	0.96 (0.72–1.26)	0.200
Absolute lymphocyte count, D3^a^	0.63 (0.45–0.95)	0.55 (0.32–0.69)	0.77 (0.51–1.11)	**0**.**005**
Absolute lymphocyte count, D6^a^	0.71 (0.46–1.02)	0.55 (0.39–0.73)	0.86 (0.65–1.20)	**<0**.**001**
Absolute lymphocyte count, D9^a^	0.89 (0.54–1.28)	0.64 (0.31–0.89)	1.27 (0.82–1.59)	**<0**.**001**

Continuous variables are presented as mean ± SD or median (IQR), and categorical variables as *n* (%).

Bold values indicate statistical significance (*P* < 0.05).

a Indicates median (IQR).

### Early trajectories of absolute lymphocyte count

**Figure 2:**
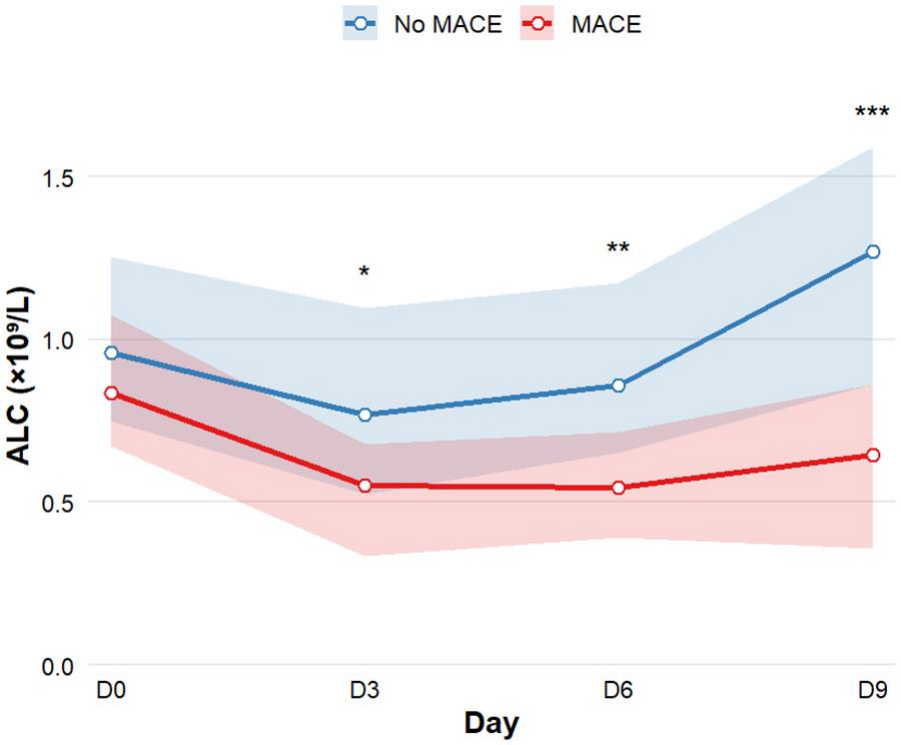
Early trajectories of absolute lymphocyte count in patients with and without major adverse cardiovascular events. Serial absolute lymphocyte count (ALC) values were measured at Day 0, Day 3, Day 6, and Day 9 after corticosteroid initiation. Solid lines represent group means, and shaded areas represent 95% confidence intervals. Between-group comparisons were performed at each time point using Bonferroni-adjusted Mann–Whitney U tests. *P < 0.05, **P < 0.01, ***P < 0.001. ALC, absolute lymphocyte count; MACE, major adverse cardiovascular events.

We tracked ALC trajectories at D0, D3, D6, and D9 for all patients. At baseline (D0), ALC did not differ between groups [0.84 (0.67–1.11) vs. 0.96 (0.72–1.26) × 10⁹/L; adjusted *p* = 0.808]. By D3, ALC was significantly lower in the MACE group than in the no-MACE group [0.55 (0.32–0.69) vs. 0.77 (0.51–1.11) × 10⁹/L; adjusted *p* = 0.019], and this difference persisted at D6 [0.55 (0.39–0.73) vs. 0.86 (0.65–1.20) × 10⁹/L; adjusted *p* = 0.002] and D9 [0.64 (0.31–0.89) vs. 1.27 (0.82–1.59) × 10⁹/L; adjusted *p* < 0.001]. Across the four time points, ALC changed significantly over time in both groups [no-MACE: *χ*²(3) = 10.7, *p* = 0.014; MACE: *χ*²(3) = 23.8, *p* < 0.001]. Pairwise *post-hoc* comparisons indicated a more pronounced early decline in the MACE group (Figure 2).

### Day-9 absolute lymphocyte count and subsequent outcomes

**Figure 3:**
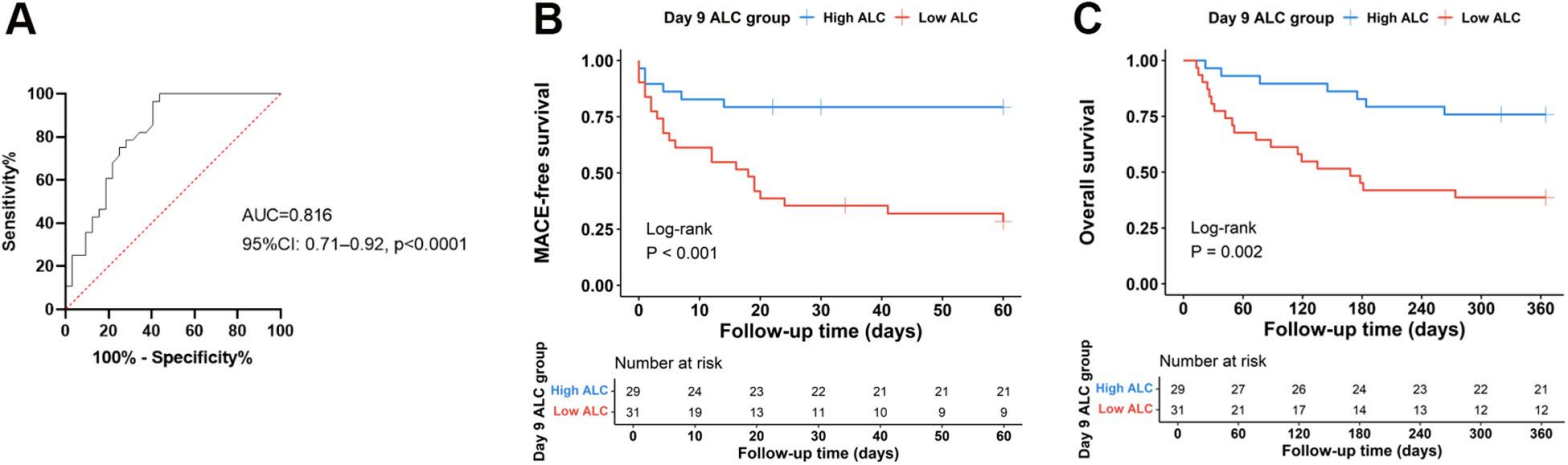
Prognostic value of Day-9 absolute lymphocyte count. **(A)** Receiver operating characteristic curve of Day-9 absolute lymphocyte count (ALC) for predicting 60-day major adverse cardiovascular events (MACE). The area under the curve was 0.816, and the optimal cutoff determined by Youden's index was 0.92 × 10⁹/L. **(B)** Kaplan–Meier curves for 60-day MACE-free survival stratified by the Day-9 ALC cutoff of 0.92 × 10⁹/L. **(C)** Kaplan–Meier curves for 1-year overall survival stratified by the same cutoff. MACE-free survival and overall survival were measured from the Day-9 landmark. Groups were compared using the log-rank test. ALC, absolute lymphocyte count; AUC, area under the curve; MACE, major adverse cardiovascular events.

ROC analysis showed that Day-9 ALC predicted MACE with good discrimination (AUC = 0.816; 95% CI, 0.71–0.92; *p* < 0.0001) (Figure 3A). Using Youden's index, the optimal cutoff was 0.92 × 10⁹/L (sensitivity 78.6%, specificity 71.9%).

Using this cutoff, Kaplan–Meier analysis demonstrated a significantly higher risk of MACE in the low-ALC group (log-rank *χ*² = 13.51, *p* < 0.001) (Figure 3B). Median MACE-free survival was 26 days in the low-ALC group, whereas the high-ALC group did not reach a median. Hazard-ratio analysis indicated that the risk of MACE in the low-ALC group was approximately 4.66-fold that of the high-ALC group (HR = 4.66; 95% CI, 1.88–11.56; *p* = 0.001).

For 1-year overall survival, Kaplan–Meier curves showed that the low-ALC group had substantially poorer survival than the high-ALC group (log-rank *χ*² = 9.36, *p* = 0.002) (Figure 3C). The median overall survival was 168 days in the low-ALC group, whereas the median was not reached in the high-ALC group. Cox proportional hazards analysis showed that the low-ALC group had a 3.57-fold higher risk of 1-year all-cause mortality than the high-ALC group (HR = 3.57; 95% CI, 1.50–8.53; *p* = 0.004). Lower Day-9 ALC was associated with a higher risk of 1-year all-cause mortality.

### Cox regression and nonlinear association between day-9 absolute lymphocyte count and MACE

In univariable Cox models (Table 3), LVEF was inversely associated with 60-day MACE (HR 0.95; 95% CI, 0.93–0.98; *p* < 0.001), while ln(NT-proBNP) was positively associated (HR 1.48; 95% CI, 1.12–1.94; *p* = 0.005). ALC on day 9 (per 1 × 10⁹/L) showed a strong inverse association with MACE (HR 0.19; 95% CI, 0.08–0.48; *p* < 0.001). Pulse-dose steroids were associated with higher risk (HR 3.81; 95% CI, 1.75–8.32; *p* < 0.001). Age and sex were not significant. In the multivariable analysis, Day-9 ALC (per 1 × 10⁹/L) remained independently associated with lower MACE risk (HR 0.33; 95% CI, 0.14–0.81; *p* = 0.016), while pulse therapy remained significant (HR 2.67; 95% CI, 1.16–6.12; *p* = 0.021).

**Table 3:** Cox regression analysis for 60-day MACE.

Variable	Univariable HR (95% CI)	*p*-value	Multivariable HR (95% CI)	*p*-value
Age (per year)	1.03 (0.98–1.07)	0.220	1.02 (0.98–1.06)	0.241
Sex = male (vs female)	1.15 (0.49–2.72)	0.743	0.83 (0.34–2.05)	0.692
LVEF (per %)	0.95 (0.93–0.98)	**<0.001**	0.98 (0.95–1.01)	0.203
ln(NT-proBNP) (per 1)	1.48 (1.12–1.94)	**0.005**	1.23 (0.89–1.70)	0.217
Day-9 ALC (per 1 × 10^9^/L)	0.19 (0.08–0.48)	**<0.001**	0.33 (0.14–0.81)	**0.016**
Steroid pulse (yes vs no)	3.81 (1.75–8.32)	**<0.001**	2.67 (1.16–6.12)	**0.021**

ALC, absolute lymphocyte count; CI, confidence interval; HR, hazard ratio; MACE, major adverse cardiovascular events.

Bold values indicate statistical significance (*P* < 0.05).

**Figure 4:**
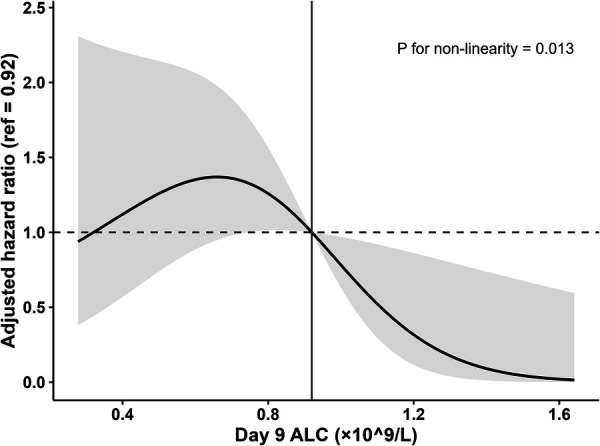
Adjusted nonlinear association between Day-9 absolute lymphocyte count and risk of 60-day major adverse cardiovascular events. Restricted cubic spline analysis was performed in a multivariable Cox proportional hazards model adjusted for age, sex, left ventricular ejection fraction, ln(NT-proBNP), and steroid pulse therapy. Day-9 absolute lymphocyte count (ALC) of 0.92 × 10⁹/L was used as the reference. The solid line represents the adjusted hazard ratio, and the shaded area represents the 95% confidence interval. ALC, absolute lymphocyte count; MACE, major adverse cardiovascular events; NT-proBNP, N-terminal pro–B-type natriuretic peptide.

To further evaluate the shape of the association, we extended the multivariable Cox model by replacing the linear Day-9 ALC term with a restricted cubic spline term, while retaining the same adjustment variables. Compared with a linear term, the spline model provided a better fit to the data (LR *χ*² = 8.67, df = 2, *p* = 0.013), with no evidence of violation of the proportional hazards assumption. Using 0.92 × 10⁹/L as the reference, lower Day-9 ALC levels were associated with a higher adjusted hazard of MACE, whereas the hazard decreased as Day-9 ALC increased (Figure 4).

### Prognostic value of absolute lymphocyte count recovery slope

Using a linear mixed-effects model, we estimated each patient's overall Day 0–9 ALC recovery slope and entered the subject-specific slope into multivariable Cox models. The overall ALC recovery slope was independently associated with lower 60-day MACE risk. When scaled per 0.1 × 10⁹/L/day, the hazard ratio was 0.07 (95% CI, 0.01–0.44; *p* = 0.004), indicating that faster ALC recovery was associated with lower subsequent MACE risk. In piecewise-slope analyses, the Day 6–9 ALC slope was significantly associated with lower MACE risk (HR 0.67 per 0.1 × 10⁹/L/day; 95% CI, 0.49–0.92; *p* = 0.012), whereas the Day 0–3 and Day 3–6 slopes did not reach statistical significance (*p* = 0.097 and *p* = 0.147, respectively) (Table 4).

**Table 4:** Multivariable cox regression analysis of ALC recovery slope for 60-day MACE.

Model	Variable	HR (95% CI)	*p*-value
Overall slope model	Age (per year)	1.02 (0.99–1.06)	0.222
	Sex = male (vs female)	0.86 (0.35–2.11)	0.745
	LVEF (per %)	0.97 (0.95–1.01)	0.102
	ln(NT-proBNP) (per 1)	1.18 (0.83–1.69)	0.350
	Steroid pulse (yes vs no)	2.45 (1.05–5.72)	**0.038**
	Overall ALC recovery slope (per 0.1 × 10^9^/L/day)	0.07 (0.01–0.44)	**0.004**
Piecewise-slope model*	D0–3 ALC slope (per 0.1 × 10^9^/L/day)	0.74 (0.52–1.06)	0.097
	D3–6 ALC slope (per 0.1 × 10^9^/L/day)	0.72 (0.46–1.12)	0.147
	D6–9 ALC slope (per 0.1 × 10^9^/L/day)	0.67 (0.49–0.92)	**0.012**

Bold values indicate statistical significance (*p* < 0.05).

*Each slope was entered separately into a multivariable Cox model adjusted for age, sex, EF, steroid pulse therapy, and ln(NT-proBNP). ALC, absolute lymphocyte count; CI, confidence interval; HR, hazard ratio; MACE, major adverse cardiovascular events.

**Figure 5:**
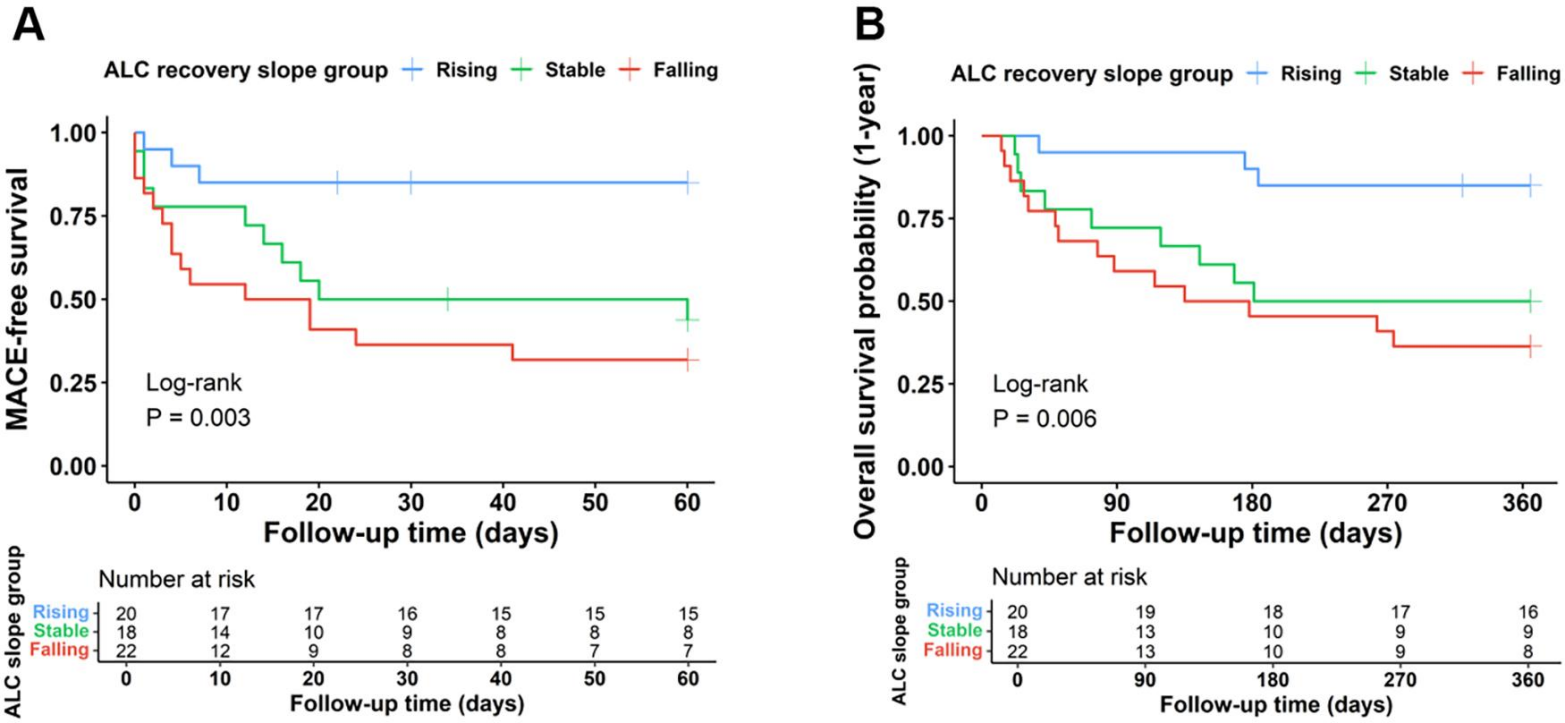
Prognostic value of early absolute lymphocyte count recovery slope. Patients were classified as Falling, Stable, or Rising according to the overall Day 0–9 absolute lymphocyte count (ALC) recovery slope using a prespecified equivalence band of ±0.01 × 10⁹/L/day. **(A)** Kaplan–Meier curves for 60-day MACE-free survival according to ALC recovery slope category. **(B)** Kaplan–Meier curves for 1-year overall survival according to ALC recovery slope category. MACE-free survival and overall survival were measured from the Day-9 landmark. Groups were compared using the log-rank test. ALC, absolute lymphocyte count; MACE, major adverse cardiovascular events.

Using a prespecified fixed equivalence band of ±0.01 × 10⁹/L/day, patients were classified into Falling, Stable, and Rising groups according to their overall Day 0–9 ALC change rate. Kaplan–Meier analysis showed significant separation among the three groups (log-rank *χ*² = 11.339, df = 2, *p* = 0.003), with a monotonic gradient of decreasing MACE risk as the ALC recovery slope increased (Figure 5A). In pairwise comparisons with Benjamini–Hochberg adjustment, the Rising group had a significantly lower risk than both the Falling group (*p* = 0.002) and the Stable group (*p* = 0.018), whereas the difference between the Stable and Falling groups was not statistically significant (*p* = 0.374).

Kaplan–Meier analysis for 1-year overall survival also showed significant separation among the three groups (log-rank *p* = 0.005), with a monotonic gradient of improving survival as the ALC recovery slope increased (Figure 5B). Pairwise Benjamini–Hochberg-adjusted comparisons showed significant differences for Rising versus Falling (*p* = 0.003) and Rising versus Stable (*p* = 0.022), but not for Stable versus Falling (*p* = 0.402). These findings were consistent with the multivariable Cox analyses, supporting that faster ALC recovery was associated with better 1-year overall survival.

## Discussion

In this study of patients with ICIAM, we identified three main results. First, a higher Day-9 ALC was associated with a lower risk of major adverse cardiovascular events, and this association was better characterized as nonlinear rather than purely linear. Second, early ALC recovery was likewise associated with a lower risk of adverse outcomes. Third, these associations remained evident after consideration of corticosteroid exposure, suggesting that the prognostic value of lymphocyte dynamics may not be explained solely by treatment-related hematologic effects.

The ALC during the early treatment course carried clinically meaningful prognostic information. Prior studies have largely emphasized diagnosis, baseline biomarkers, or acute cardiac manifestations, whereas less attention has been given to hematologic parameters measured during the early treatment course (6, 10, 12, 13). Drobni et al. reported that lymphocyte-related parameters, including decreased ALC and increased neutrophil-to-lymphocyte ratio, were associated with subsequent MACE in patients with ICIAM, less attention has been given to serial hematologic changes during the early treatment course. Our study extends this prior evidence by showing that Day-9 ALC and early ALC recovery after corticosteroid initiation were associated with subsequent adverse outcomes. In our cohort, higher Day-9 ALC was associated with lower MACE risk, and spline modeling suggested that this relationship was nonlinear, with only modest risk separation in the lower ALC range but a steeper decline in risk once Day-9 ALC reached a higher level. This pattern may indicate a clinically relevant threshold-like effect. A plausible explanation is that low peripheral ALC may partly reflect recruitment or redistribution of activated T cells into inflamed myocardium. This interpretation is supported by pathological studies showing patchy myocardial infiltrates rich in CD4 + and CD8+ T cells in ICIAM, with greater T-cell infiltration in more fulminant cases (21, 22). Paired heart-blood immune profiling has further shown enrichment of cytotoxic T cells in the myocardium together with circulating T-cell populations sharing heart-expanded clonotypes, supporting a biologic link between peripheral immune dynamics and cardiac inflammation (23). In addition, recent translational research implicates the CXCR3-CXCL9/CXCL10 axis in T-cell recruitment to the heart (24).

Early lymphocyte recovery was also associated with lower risk of adverse outcomes. Biologically, delayed recovery may reflect persistent immune dysregulation, ongoing myocardial inflammation, or incomplete resolution of the acute disease process (16, 23, 25). Clinically, this early period is especially relevant because decisions regarding monitoring intensity and treatment escalation often need to be made during the first days after presentation (6, 26). A broadly similar prognostic signal has been reported in other critical illnesses, particularly sepsis and severe pneumonia, in which persistent lymphopenia or lower lymphocyte counts have been associated with greater illness severity and worse short-term outcomes (27, 28). Taken together, these observations support the possibility that recovery of peripheral lymphocyte counts reflects restoration of immune homeostasis and is therefore associated with more favorable clinical outcomes.

Glucocorticoid exposure is an important consideration when interpreting dynamic lymphocyte counts in ICIAM. Corticosteroids are the cornerstone of first-line treatment and can substantially alter peripheral lymphocyte counts through lymphocyte redistribution and immunomodulatory effects (29, 30). In addition, patients with more severe disease are often more likely to receive intensive steroid treatment, creating potential confounding by indication (30). To mitigate this bias, we assessed steroid exposure across prespecified treatment windows and used a landmark approach to evaluate subsequent risk among patients who remained event-free to that time point. Classical human and experimental studies have shown that steroid-induced lymphocytopenia is often transient, with an early decline in circulating lymphocytes followed by recovery within 24 h; with higher-dose systemic hydrocortisone, rebound above baseline may even occur (31–33). In our cohort, the no-MACE group broadly followed this expected steroid-related pattern, whereas patients with MACE remained persistently lymphopenic despite more intensive early corticosteroid exposure. Because steroid doses were not significantly different between groups during later tapering, these findings argue against corticosteroid exposure alone as the main explanation for the separated trajectories and instead suggest that low Day-9 ALC and impaired early lymphocyte recovery more likely reflect greater disease severity and persistent immune dysregulation.

It is also important to interpret ALC dynamics in the context of established cardiac markers and pre-existing cardiovascular vulnerability (34–36). In the present study, lower LVEF and higher ln(NT-proBNP) were associated with MACE in univariable analyses, consistent with their roles as markers of myocardial involvement, hemodynamic stress, and overall disease severity. Their attenuation after multivariable adjustment should therefore not be interpreted as evidence of limited clinical relevance. Rather, LVEF, NT-proBNP, and corticosteroid pulse therapy may partly represent overlapping dimensions of clinical severity, because patients with more aggravated presentations are more likely to have impaired cardiac function, elevated natriuretic peptide levels, and receive more intensive corticosteroid treatment. And ALC is a non-specific biomarker and may be influenced by factors including nutritional status, infection, systemic inflammation and tumor burden. Although baseline laboratory parameters including albumin, WBC, and neutrophil count were evaluated, residual confounding by unmeasured or incompletely captured factors cannot be excluded. Accordingly, Day-9 ALC and early lymphocyte recovery should be viewed as complementary immune-inflammatory markers rather than substitutes for established cardiac biomarkers, imaging findings, or clinical risk factors.

From a clinical perspective, these findings suggest that early lymphocyte dynamics may provide a simple and pragmatic approach for risk stratification in patients with ICIAM. Because absolute lymphocyte count is routinely available and easily monitored, incorporation of Day-9 ALC and early recovery patterns into clinical assessment may help identify patients at higher risk of adverse cardiovascular events and mortality. Such patients may benefit from closer monitoring and timely multidisciplinary reassessment of treatment strategy. If validated in larger cohorts, this approach may complement existing biomarker and imaging-based strategies and help refine multidisciplinary management of this high-risk population.

### Limitations

Several limitations should be acknowledged. First, this was a retrospective observational study, and residual confounding cannot be fully excluded. Second, the sample size was limited, which may reduce statistical precision, particularly for subgroup and nonlinear analyses. And not all patients underwent CMR or endomyocardial biopsy. Some cases were clinically diagnosed. Therefore, potential diagnostic misclassification cannot be completely excluded, particularly in patients without CMR or EMB confirmation. Third, corticosteroid exposure remains a major source of complexity when interpreting serial lymphocyte counts, and complete separation of treatment effects from disease biology is not possible in an observational cohort. Fourth, ALC is a nonspecific immune marker and may be influenced by infection, cancer status, concomitant therapies, and other systemic conditions. Fifth, ALC trajectories appeared to continue diverging after Day 9, but later values were not analyzed because of increasing risks of informative missingness and survivor bias. Sixth, this design may have introduced selection bias by excluding patients with insufficient early clinical records or incomplete early follow-up. Therefore, the findings should be interpreted as hypothesis-generating and require validation in further study. Finally, external validation is needed before these findings can be generalized to broader populations.

## Conclusions

In patients with ICIAM, lower Day-9 ALC and slower lymphocyte recovery were associated with worse clinical outcomes. Day-9 ALC provides early prognostic information for short-term cardiovascular events, and dynamic ALC recovery patterns further improved risk stratification. These findings support the potential value of serial ALC assessment as a simple and clinically accessible tool for early prognostic evaluation in ICIAM.

## Data Availability

The original contributions presented in the study are included in the article/Supplementary Material, further inquiries can be directed to the corresponding author.

## References

[1] Mahmood SS Fradley MG Cohen JV Nohria A Reynolds KL Heinzerling LM. Myocarditis in patients treated with immune checkpoint inhibitors. J Am Coll Cardiol. (2018) 71:1755–64. 10.1016/j.jacc.2018.02.037 29567210 PMC6196725

[2] Moslehi J Lichtman AH Sharpe AH Galluzzi L Kitsis RN. Immune checkpoint inhibitor–associated myocarditis: manifestations and mechanisms. J Clin Invest. (2021) 131:e145186. 10.1172/JCI145186 33645548 PMC7919710

[3] Xu Y Song Y Liu X Shi Y Liu Y Qian H. Prediction of major adverse cardiac events is the first critical task in the management of immune checkpoint inhibitor-associated myocarditis. Cancer Commun. (2022) 42:902–5. 10.1002/cac2.12320 PMC9456696 35678260

[4] Frascaro F Bianchi N Sanguettoli F Marchini F Meossi S Zanarelli L. Immune checkpoint inhibitors-associated myocarditis: diagnosis, treatment and current Status on rechallenge. J Clin Med. (2023) 12:7737. 10.3390/jcm12247737 38137806 PMC10744238

[5] Turker I Johnson DB. Immune checkpoint inhibitor-related myocarditis: current understanding and potential diagnostic and therapeutic strategies. Expert Opin Drug Saf. (2023) 22:909–19. 10.1080/14740338.2023.2254218 37647330 PMC10530188

[6] Palaskas N Lopez-Mattei J Durand JB Iliescu C Deswal A. Immune checkpoint inhibitor myocarditis: pathophysiological characteristics, diagnosis, and treatment. J Am Heart Assoc. (2020) 9:e013757. 10.1161/JAHA.119.013757 31960755 PMC7033840

[7] Arcari L Tini G Camastra G Ciolina F De Santis D Russo D. Cardiac magnetic resonance imaging in immune check-point inhibitor myocarditis: a systematic review. J Imaging. (2022) 8:99. 10.3390/jimaging8040099 35448226 PMC9027245

[8] Pereyra Pietri M Farina JM Awad K Kanaan CN Scalia IG Tagle-Cornell C. Diagnostic and prognostic value of high-sensitivity troponin T for cardiovascular outcomes in patients receiving immune checkpoint inhibitor therapy. J Am Heart Assoc. (2026) 15:e041680. 10.1161/JAHA.125.041680 41294120 PMC13055820

[9] Zhou K Liu G Zhang B Zhang X Wu W. Electrocardiographic predictors of major adverse cardiovascular events in immune checkpoint inhibitor-associated myocarditis. Cardio-Oncol. (2026) 12:42. 10.1186/s40959-026-00455-5 PMC13032447 41715261

[10] Murtagh G Defilippi C Zhao Q Barac A. Circulating biomarkers in the diagnosis and prognosis of immune checkpoint inhibitor-related myocarditis: time for a risk-based approach. Front Cardiovasc Med. (2024) 11:1350585. 10.3389/fcvm.2024.1350585 38410245 PMC10894940

[11] Fan Y Xu Y Liu X Liu G Zhang B Wu W. Immune checkpoint inhibitor-related myocarditis: a comprehensive analysis of clinical manifestations and prognostic factors. Oncologist. (2025) 30:oyaf285. 10.1093/oncolo/oyaf285 40996334 PMC12808996

[12] Drobni ZD Zafar A Zubiri L Zlotoff DA Alvi RM Lee C. Decreased absolute lymphocyte count and increased neutrophil/lymphocyte ratio with immune checkpoint inhibitor-associated myocarditis. J Am Heart Assoc. (2020) 9:e018306. 10.1161/JAHA.120.018306 33190570 PMC7763791

[13] Dubey N Wu C-Y Zubiri L Fay M Rouhani SJ Merkin RD. Predictors of long-term survival in patients with immune checkpoint inhibitor-associated myocarditis. J Am Heart Assoc. (2025) 14:e038719. 10.1161/JAHA.124.038719 40611488 PMC12533668

[14] Schulz-Menger J Collini V Gröschel J Adler Y Brucato A Christian V. 2025 ESC guidelines for the management of myocarditis and pericarditis. Eur Heart J. (2025) 46:3952–4041. 10.1093/eurheartj/ehaf192 40878297

[15] Palaskas NL Siddiqui BA Deswal A. Steroids in immune checkpoint inhibitor myocarditis. JACC CardioOncology. (2024) 6:800–3. 10.1016/j.jaccao.2024.07.002 39479336 PMC11520217

[16] Feng S Zhao B Sha S Bu X Zhang Z Liu G. Overview of immune checkpoint inhibitor associated myocarditis mechanisms diagnostics and treatment. Front Immunol. (2025) 16:1677984. 10.3389/fimmu.2025.1677984 41394853 PMC12698404

[17] Zhang L Zlotoff DA Awadalla M Mahmood SS Nohria A Hassan MZO. Major adverse cardiovascular events and the timing and dose of corticosteroids in immune checkpoint inhibitor-associated myocarditis. Circulation. (2020) 141:2031–4. 10.1161/CIRCULATIONAHA.119.044703 32539614 PMC7301778

[18] Lyon AR López-Fernández T Couch LS Asteggiano R Aznar MC Bergler-Klein J. 2022 ESC guidelines on cardio-oncology developed in collaboration with the European Hematology Association (EHA), the European Society for Therapeutic Radiology and Oncology (ESTRO) and the International Cardio-Oncology Society (IC-OS). Eur Heart J. (2022) 43:4229–361. 10.1093/eurheartj/ehac244 36017568

[19] Ferreira VM Schulz-Menger J Holmvang G Kramer CM Carbone I Sechtem U. Cardiovascular magnetic resonance in nonischemic myocardial inflammation: expert recommendations. J Am Coll Cardiol. (2018) 72:3158–76. 10.1016/j.jacc.2018.09.072 30545455

[20] Aretz H T. Myocarditis: the Dallas criteria. Hum Pathol. (1987) 18:619–24. 10.1016/S0046-8177(87)80363-5 3297992

[21] Champion SN Stone JR. Immune checkpoint inhibitor associated myocarditis occurs in both high-grade and low-grade forms. Mod Pathol Off J U S Can Acad Pathol Inc. (2020) 33:99–108. 10.1038/s41379-019-0363-0 31534205

[22] Sobol I Chen CL Mahmood SS Borczuk AC. Histopathologic characterization of myocarditis associated with immune checkpoint inhibitor therapy. Arch Pathol Lab Med. (2020) 144:1392–6. 10.5858/arpa.2019-0447-OA 32150459 PMC8445131

[23] Blum SM Zlotoff DA Smith NP Kernin IJ Ramesh S Zubiri L. Immune responses in checkpoint myocarditis across heart, blood and tumour. Nature. (2024) 636:215–23. 10.1038/s41586-024-08105-5 39506125 PMC12952943

[24] Huang YV Sun Y Chou H Wagner N Vitale MR Bayer AL. Novel therapeutic approach targeting CXCR3 to treat immunotherapy myocarditis. Circ Res. (2025) 136:473–90. 10.1161/CIRCRESAHA.124.325652 39931812 PMC11867805

[25] Won T Kalinoski HM Wood MK Hughes DM Jaime CM Delgado P. Cardiac myosin-specific autoimmune T cells contribute to immune-checkpoint-inhibitor-associated myocarditis. Cell Rep. (2022) 41:111611. 10.1016/j.celrep.2022.111611 36351411 PMC11108585

[26] Raschi E Rossi S De Giglio A Fusaroli M Burgazzi F Rinaldi R. Cardiovascular toxicity of immune checkpoint inhibitors: a guide for clinicians. Drug Saf. (2023) 46:819–33. 10.1007/s40264-023-01320-5 37341925 PMC10442274

[27] Drewry AM Samra N Skrupky LP Fuller BM Compton SM Hotchkiss RS. Persistent lymphopenia after diagnosis of sepsis predicts mortality. Shock Augusta Ga. (2014) 42:383–91. 10.1097/SHK.0000000000000234 25051284 PMC4362626

[28] Cilloniz C Peroni HJ Gabarrús A García-Vidal C Pericàs JM Bermejo-Martin J. Lymphopenia is associated with poor outcomes of patients with community-acquired pneumonia and sepsis. Open Forum Infect Dis. (2021) 8:ofab169. 10.1093/ofid/ofab169 34189165 PMC8231373

[29] Liberman AC Budziñski ML Sokn C Gobbini RP Steininger A Arzt E. Regulatory and mechanistic actions of glucocorticoids on T and inflammatory cells. Front Endocrinol. (2018) 9:235. 10.3389/fendo.2018.00235 PMC5964134 29867767

[30] Itzhaki Ben Zadok O Levi A Divakaran S Nohria A. Severe vs nonsevere immune checkpoint inhibitor-induced myocarditis: contemporary 1-year outcomes. JACC CardioOncology. (2023) 5:732–44. 10.1016/j.jaccao.2023.09.004 38205012 PMC10774775

[31] Fauci AS Dale DC. The effect of *in vivo* hydrocortisone on subpopulations of human lymphocytes. J Clin Invest. (1974) 53:240–6. 10.1172/JCI107544 4808638 PMC301459

[32] Olnes MJ Kotliarov Y Biancotto A Cheung F Chen J Shi R. Effects of systemically administered hydrocortisone on the human immunome. Sci Rep. (2016) 6:23002. 10.1038/srep23002 26972611 PMC4789739

[33] Bromberg L Roufosse F Pradier O Delporte C Van Antwerpen P De Maertelaer V. Methylprednisolone-induced lymphocytosis in patients with immune-mediated inflammatory disorders. Am J Med. (2016) 129:746–752.e3. 10.1016/j.amjmed.2016.02.013 26968468

[34] Pătru I-R Anghel A-V Galeschi ER Bătăuș LC Ionescu A-I Negru AG. The impact of immune-related adverse events on the survival of patients treated with immune checkpoint inhibitors: the distinct role of cardiac toxicities. J Clin Med. (2025) 14:7794. 10.3390/jcm14217794 41227189 PMC12609892

[35] Pătru I-R Ionescu A-I Negru AG Anghel A-V Iordache M Becheru OG. Predictors of immune-related cardiac adverse events in patients receiving immune checkpoint inhibitors: a retrospective cohort study. J Clin Med. (2026) 15:1763. 10.3390/jcm15051763 41827180 PMC12986019

[36] Power JR Dolladille C Ozbay B Procureur A Ederhy S Palaskas NL. Immune checkpoint inhibitor-associated myocarditis: a novel risk score. Eur Heart J. (2026) 47(9):1050–1062. 10.1093/eurheartj/ehaf315 PMC13291914 40569849

